# Assessing the Effects of Trematode Infection on Invasive Green Crabs in Eastern North America

**DOI:** 10.1371/journal.pone.0128674

**Published:** 2015-06-01

**Authors:** April M. H. Blakeslee, Carolyn L. Keogh, Amy E. Fowler, Blaine D. Griffen

**Affiliations:** 1 Long Island University, 720 Northern Blvd., Brookville, NY, 11548, United States of America; 2 Smithsonian Environmental Research Center, 647 Contees Wharf Rd., Edgewater, MD, 21037, United States of America; 3 University of Georgia, 140 E. Green St., Athens, GA, 30602, United States of America; 4 Marine Resources Research Institute, South Carolina Department of Natural Resources, 217 Fort Johnson Road, Charleston, SC, 29422, United States of America; 5 University of South Carolina, Department of Biological Sciences and Marine Science Program, 715 Sumter Street, Columbia, SC, 29208, United States of America; National University of Singapore, SINGAPORE

## Abstract

A common signature of marine invasions worldwide is a significant loss of parasites (= parasite escape) in non-native host populations, which may confer a release from some of the harmful effects of parasitism (e.g., castration, energy extraction, immune activation, behavioral manipulation) and possibly enhance the success of non-indigenous species. In eastern North America, the notorious invader *Carcinus maenas* (European green crab) has escaped more than two-thirds its native parasite load. However, one of its parasites, a trematode (*Microphallus similis*), can be highly prevalent in the non-native region; yet little is known about its potential impacts. We employed a series of laboratory experiments to determine whether and how *M*. *similis* infection intensity influences *C*. *maenas*, focusing on physiological assays of body mass index, energy storage, and immune activation, as well as behavioral analyses of foraging, shelter utilization, and conspicuousness. We found little evidence for enduring physiological or behavioral impacts four weeks after experimental infection, with the exception of mussel handling time which positively correlated with cyst intensity. However, we did find evidence for a short-term effect of *M*. *similis* infection during early stages of infection (soon after cercarial penetration) via a significant drop in circulating immune cells, and a significant increase in the crabs’ righting response time. Considering *M*. *similis* is the only common parasite infecting *C*. *maenas* in eastern North America, our results for minimal lasting effects of the trematode on the crab’s physiology and behavior may help explain the crab’s continued prominence as a strong predator and competitor in the region.

## Introduction

Over the past few centuries, human activities have transported numerous marine organisms to foreign coastlines at an increasing rate as global trade and shipping methods have become more efficient and widespread [1–4]. Accidental or intentional introductions of marine non-indigenous species (NIS) have resulted in the addition of numerous new organisms, including free-living and parasitic hitchhikers, to marine environments around the world. Once a species has become established and spreads within a foreign environment, it may induce extensive change to its recipient community; for example, NIS have been shown to modify habitats, cause shifts in trophic dynamics (e.g., trophic cascades), and alter community composition and structure [5–12].

A commonly cited factor contributing to the success of many NIS in recipient communities is their release from coevolved natural enemies, including predators, competitors, and parasites [13–16]. Specific to parasites, reductions in the diversity and intensity of parasite infections in non-native hosts is a strong signature of marine invasions globally, and on average, parasite loads in non-indigenous populations are approximately half that of native populations [17]. Such significant parasite losses may benefit host survival and reproduction if hosts are released from the many harmful effects of parasite infection, such as castration, enhanced mortality, energy extraction, immune activation, and/or behavioral modifications [14, 16–18]. However, the benefit of parasite release may be temporary if parasites from the host’s native range invade with subsequent introduction events, or hosts acquire new generalist parasites in their recipient communities [14]. For example, in the 1950s the Red Sea swimming crab *Charybdis longicollis* invaded the Mediterranean Sea via the Suez Canal, escaping a castrating rhizocephalan parasite in the process; however, in 1992 the parasite also invaded the region, thereby eliminating the crab’s temporary release from parasitic castration [19–21].

**Fig 1:**
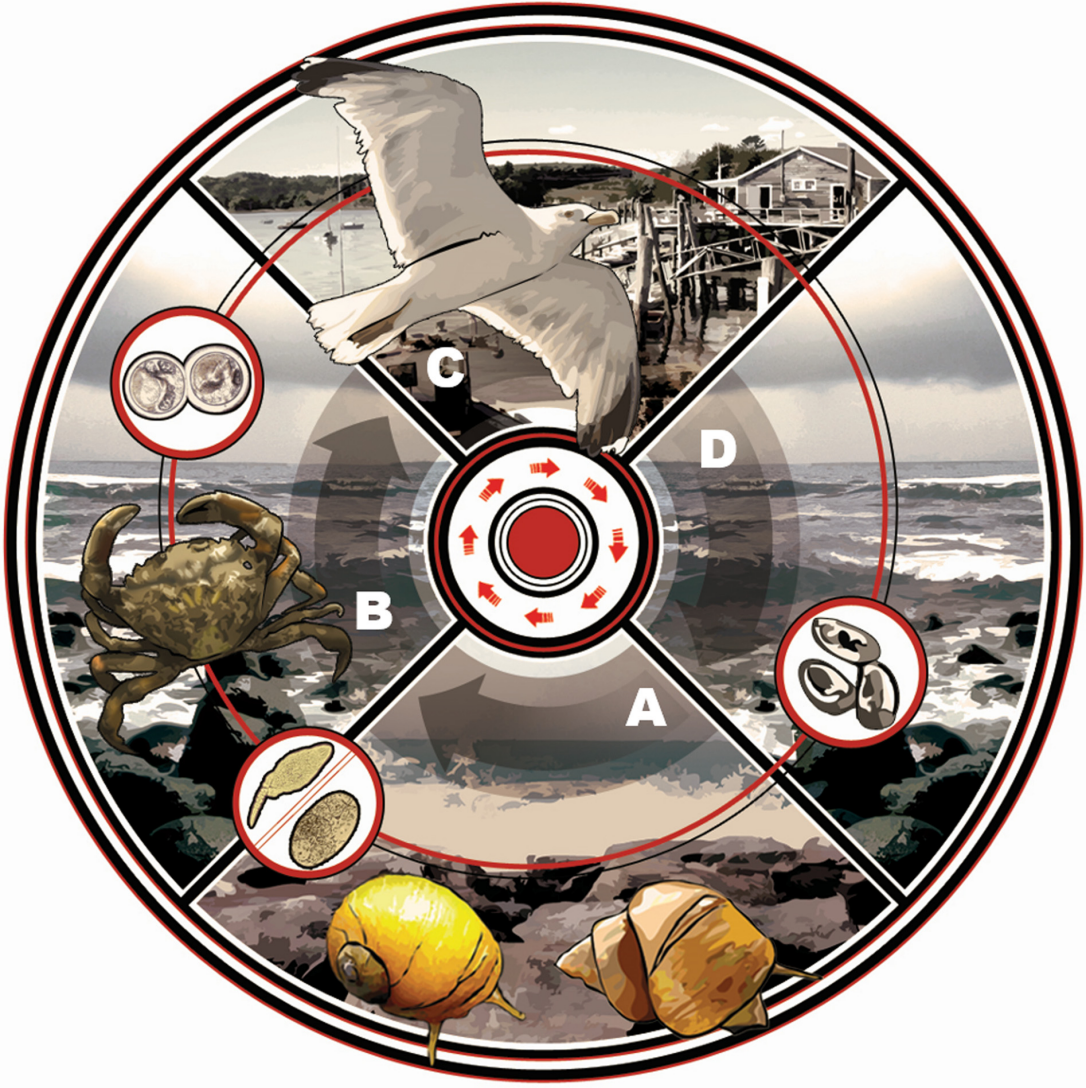
Life cycle of *Microphallus similis* in eastern North America with *Carcinus maenas* as second-intermediate host. *Microphallus similis* infects multiple hosts to complete its life cycle, starting with (A) two species of *Littorina* snails (*L*. *saxatilis* and *L*. *obtusata*), where the trematode asexually reproduces, producing numerous cercariae. (B) These cercariae are shed from the snail into the water column, where they seek out and encyst as a metacercariae within a second-intermediate host, primarily the green crab, *Carcinus maenas*. To sexually reproduce, (C) the trematode’s crab host must be ingested by a definitive host, often a *Larus* gull species, where (D) the trematode’s eggs, containing miracidia, are then deposited into the marine environment with the birds’ feces. Grazing snails accidentally ingest these eggs, and the cycle continues.

The most frequently observed parasitic taxa infecting marine NIS worldwide are digenean trematodes [17], a group known to modify host behaviors and physiology and consequently influence population and community level dynamics [22]. Trematodes use multi-host trophic transmission to complete life cycles, infecting numerous species across a broad phylogenetic spectrum [23–24]. First-intermediate hosts are typically gastropods, which are castrated as the trematode asexually reproduces in the gonad region, generating copious cercariae that are continually shed from the snail. Cercariae seek and encyst as metacercariae within second-intermediate hosts (crustaceans, fish, gastropods, bivalves, worms) and remain dormant until ingestion by the definitive host (fish, birds, mammals). Some trematode species have a truncated life cycle and emerging cercariae directly penetrate definitive hosts, or cercariae encyst on hard/firm substrates (e.g., shell or algae) that are ingested by definitive hosts. Sexual reproduction occurs in the definitive host’s gut, and thousands of trematode eggs enter the marine system via host defecation (Fig 1).

Trematodes exert their strongest physiological and behavioral effects on intermediate hosts, and their influences can range from relatively minor to extreme [24–25]. Physiological effects can include castration, impaired reproductive function, tissue damage, immune activation, energetic diversion of host resources, and tissue or organ dysfunction [26–32]. For example, metacercarial encystment in second-intermediate hosts has been shown to compress and rupture digestive gland (hepatopancreas) tubules [33], induce hemocytic infiltration around cysts [34], and lower host growth rates [35]. Behavioral impacts can include manipulations of host foraging, shelter use, predator avoidance, and conspicuousness. For example, infected hosts have been shown to compensate for the metabolic stress of trematode infection by increasing foraging rates [25, 36], or they may reduce foraging if mobility or prey handling become compromised [37–38]. Trematodes can also enhance trophic transmission by altering predator avoidance behaviors (e.g., modifying shelter usage) [39] or inducing behavioral alterations such that intermediate hosts are more conspicuous to vertebrate predators, thereby increasing the likelihood of transmission to, and sexual reproduction within, definitive hosts (= parasite increased trophic transmission) [25, 40–41]. A well-known example involves the Pacific killifish, which is infected by a trematode that concentrates in the fish’s brain and induces highly conspicuous behaviors like “flashing, surfacing, contorting, shimmying, and jerking”; in turn, infected killifish are preyed upon by seabird definitive hosts at significantly higher rates than are uninfected fish [42].

With such diverse effects on host physiology and behavior, trematodes have the potential to influence NIS in regions where hosts are infected by prevalent hitchhiking or newly acquired trematodes. Here, we focus on non-indigenous populations of the European green crab *Carcinus maenas*, one of the most infamous marine invaders globally and a common host to numerous parasite species including trematodes [18, 43–44]. *Carcinus maenas* has been introduced to five continents and is implicated in significant predatory and competitive impacts on numerous intertidal and subtidal habitats and organisms, including commercial species like clams, scallops, and mussels [5, 45–47]. In the crab’s oldest introduced region in eastern North America (established for ~200 years; [43]), *C*. *maenas* has escaped 70% of its native parasite load [44], and crabs in the region can attain larger average sizes, are more intact (reduced limb loss), and are healthier overall than in native populations [18]—likely due in part to the crab’s escape from castrating *Sacculina* spp. rhizocephalan barnacles [48–50]. The few parasite species that do infect *C*. *maenas* in eastern North America include an acanthocephalan, a larval nematode, and the trematode *Microphallus similis;* the first two species are relatively uncommon, but *M*. *similis* can be highly prevalent in the region (up to 100% in adult crabs in some populations) [44]. *Microphallus similis* may have hitchhiked to North America with *C*. *maenas* during the crab’s multiple introductions to the region (consisting of at least two major events; [51]), or perhaps it was acquired from native *Littorina spp*. periwinkle snails that serve as first-intermediate hosts [44]. The trematode has a three-host infection cycle with a larval cercarial stage in periwinkles, a metacercarial stage in *C*. *maenas*, and a final adult stage in shorebirds (Fig 1). Though infected *C*. *maenas* may harbor hundreds to thousands of *M*. *similis* metacercarial cysts in hepatopancreas and gonad tissues, nothing is yet known about potential influences on *C*. *maenas*. Understanding impacts is important because any discernible trematode-modified behaviors and/or physiology could ultimately affect the crab’s interactions in coastal North American communities, such as its predation rates on intertidal and subtidal organisms [52], its ability to effectively compete with other abundant shorecrabs like the non-native Asian shorecrab, *Hemigrapsus sanguineus* [44, 53–54], or its predation by larger predators like shorebirds [55].

In our study, we use a series of laboratory experiments and field observations to determine whether and how *M*. *similis* infection intensity influences *C*. *maenas* physiology and/or behavior. Related to physiology, we investigate whether *M*. *similis* affects the mass and energy content of hepatopancreas and gonad tissues in the crab and whether the parasite stimulates immune activation via hemolymph cell counts. We also assess whether infected individuals exhibit behavioral response to infection, particularly focusing on consumption, shelter utilization, and conspicuous/active behaviors that could influence the crabs’ predation risk. Our results provide a baseline understanding of trematode-induced effects on this influential crab species in its oldest invaded range.

## Methods

### Infection Induction Experiment

In June 2012, we collected 72 male (n = 35) and female (n = 37) *Carcinus maenas* ranging from 45–70 (AVG ± SD: 59.34 ± 7.04) mm carapace width (CW) from Adams Point, Durham, NH, a site with naturally low trematode infection prevalence in both snail and crab hosts (<0.05%; [44, 56]). Field collections of green crabs at this site did not require specific permission, and green crabs are not an endangered species (they are a non-native, nuisance species in eastern North America). Live crabs were transported to the Shoals Marine Laboratory (SML) on Appledore Island (ME), where they were divided evenly by sex and size into three exposure and three control groups (n = 12 crabs per group). Each group was placed into a ~50 liter clear plastic aquarium containing ~20 liters of aerated seawater. Since metacercarial cyst infection intensity has been shown to positively correlate with physiological and behavioral effects in intermediate hosts (e.g., [42]), we induced a gradient of *Microphallus similis* infection in experimental groups by exposing them to trematode cercariae shed from naturally infected first-intermediate snail hosts, *Littorina saxatilis* and *L*. *obtusata*, for either 24, 72, or 120 hours. These periwinkle snails (n = 1800; >8mm shell length; AVG ± SD: 10.80 ± 1.66) were collected (under an annual collecting permit issued to SML by the state of Maine) from a relatively high trematode infection site on Appledore Island [57] and were divided into two batches (Batch-1 & Batch-2, n = 900 snails each) to induce cercarial emergence (shedding) via dessication ([23], pers. obs.). Snails were alternately dessicated every 24 hours, and then batches were pooled and randomly divided into three groups to spread out infected snails across experimental treatments. 300 of these snails were placed in each aquarium in a metal strainer covered with fine mesh and suspended at the top of the water column to contain and protect snails but also allow shed cercariae to swim downwards to crabs [58]. The three control aquaria contained empty metal strainers. Cercarial emergence was enumerated twice daily (0800h and 2000h) by pooling five 1-mL water samples from the four corners and center of the container and systematically scanning for cercariae under a stereomicroscope at 4x power (10x oculars) (S1 Fig). After morning cercarial counts, 50% water changes were performed. Following their allotted exposure times, crabs were removed from treatment aquaria, weighed and assessed for missing limbs. To eliminate cannibalism risk, crabs were placed into individual plastic aquaria (9.13"L x 6.00"W x 6.12"H) and submerged in a large flow-through sea table at SML for a four week ‘incubation’ period, which provided enough time for metacercarial cysts to reach patency and demonstrate a distinct cyst wall ([59], pers. obs.). During this time, crabs were fed locally-collected *Ulva* spp. *ad libitum*. This diet was chosen because it provided limited but sufficient resources for all crabs; moreover, we expected a non-animal diet would enhance detectable differences among treatments when mussels were later added during behavioral trials (see below) since crabs had been deprived of animal protein for four weeks.

### Righting Response Trials

Two hours after the induction experiment described above, we performed righting response trials in a subset (n = 11; 5 exposed and 6 control) of the 72-h exposure group to explore whether infected crabs would differ in their ability to right themselves compared to uninfected crabs shortly after cercarial penetration of their tissues. For these trials, individual crabs were removed from the treatment aquaria, immediately placed dorsal side down in a ~40 liter container, and timed for how long it took them to flip back over or right themselves. Each crab was tested three times for an average righting time, and trials were suspended if a crab had not righted itself within five minutes. Results of average righting response times in infected versus uninfected crabs were analyzed using a t-test. This analysis and all subsequent statistical analyses were performed using JMP 9.0.2 (SAS Institute).

### Behavioral Trials and Video Analysis

Behavior trials were conducted to explore whether infection intensity influenced crab behaviors related to foraging, shelter utilization, and/or conspicuousness. Following their 4-week ‘incubation’ period, 70 crabs (35 exposed, 35 control) were examined for behavior around the evening high tide (when crabs are often the most active; [60–62]). Experiments were carried out in experimental arenas (S2 Fig), consisting of two replicate ~40 liter plastic containers wrapped in black plastic (to limit light exposure) and filled with ~20 liters of aerated seawater. Crabs were paired so that an experimental trial and its control (e.g., 24-h exposed and 24-h control) were run at the same time. To simulate a natural shelter, a single rock standardized for size and dimensions was covered with 83g of *Fucus vesiculosis* attached using rubber-bands. *Fucus* fronds were unique for every trial, and rocks were reused after a thorough washing and drying outside for 24h. Shelters were placed at randomly alternated ends of the arena. Before each trial, crabs were acclimated to the arenas for 30 minutes. Following the acclimation period, five live individually-marked mussels ranging from 12-20mm (16.68 ± 2.41mm) shell length were added to the opposite end of the container from the shelter. Video cameras were mounted above the arenas, which were illuminated using red lighting, and crab behaviors were recorded for 30 minutes. Ten crabs were assessed each evening over a 7-day period. Water was changed completely between trials.

Each crab video (n = 68 due to two corrupted videos) was assessed by a single observer (AMHB). An ethogram (S1 File) was used to collect behavioral data every 30s for 30m (n = 60 data points) to provide a relative understanding of the proportion of time and the total amount of time (in seconds) crabs spent doing specific behaviors during the recording period. The recorded behaviors included active behaviors (walking, climbing on shelter, climbing up aquaria walls, actively handling or consuming mussels) and inactive behaviors (standing still, under shelter, on shelter, standing next to shelter). Each behavior was regressed with cyst abundance to look for correlations between infection intensity and the particular behavior, or a larger grouping of behaviors (e.g., active, inactive). We also regressed temporal data (in seconds) for three variables (search time for mussels, time spent in association with shelter, and handling time for each mussel and all mussels collectively) with cyst abundance. Finally, we regressed the total number of mussels eaten per crab at the end of the 30m period with cyst abundance to determine if infection intensity influenced mussel consumption.

### Crab Dissections and Cyst Counts

Crabs were dissected the morning after their individual behavioral trials using protocols modified from [18, 44, 63]. Crabs were anaesthetized by freezing for 30m at 0°C and then dissected by separating the upper and lower carapace using a sterilized razor blade. A series of 10 tissue squashes—i.e., the amount of tissue needed to fill the area of a 22x22-mm glass cover slip—were removed from each crab to capture a large subset of the hepatopancreas (8 squashes), thoracic ganglion (1 squash), and gonad (1 squash). Squashes focused on the hepatopancreas because prior work found cysts to concentrate in this area [18, 44, 63]. Each squash slide was weighed and then viewed under a compound microscope at 4x (10x oculars) to enumerate all *M*. *similis* metacercarial cysts per squash using a handheld tally counter. Experimentally induced (new) cysts were easily differentiated from naturally acquired (old) cysts based on the presence of a thick cyst wall in old cysts as opposed to a very thin cyst wall in new cysts ([59]; S3 Fig). We rarely found old cysts in dissected crabs (<0.05% of all counted cysts), demonstrating very low initial metacercarial infection in crabs. Following cyst counts, all hepatopancreas and gonad tissues (including those scraped from slides) were removed from each crab and kept frozen for later physiological analyses (see below). In one heavily infected crab (>7000 counted cysts), we also took several photographs (n = 7) to estimate the percentage of internal space cysts may take up in an infected crab’s hepatopancreas (using ImageJ analysis). Finally, we gathered cyst intensity data from crabs collected at a local SML field site in 2012 (n = 4) and 2007 (n = 27) (the latter reported in [44]) to compare cyst intensities between experimentally-infected crabs in the lab and naturally infected crabs in the field. Total cyst abundance in naturally infected and experimentally infected crabs in each of the exposure treatments (controls, 24-h, 72-h, 120-h) were compared in a one-way ANOVA to test for differences in cyst intensity among the treatment groups.

### Hepatosomatic and Gonadosomatic Indices and Estimation of Total Cyst Abundance and Mass

We separately dried the hepatopancreas, the gonads (male or female), and the remainder of the body of each crab (including the four naturally infected crabs from Appledore Island) at 70°C for 72h. Each component was then weighed using a digital scale. The hepatosomatic index (HSI) and the gonadosomatic index (GSI) were calculated as the ratio of dried hepatopancreas or gonad to the overall dried body weight of a crab [54] after subtracting off any metacercarial cyst mass. To determine metacercarial mass in the hepatopancreas, we used cyst abundance and hepatopancreas squash weights (g) to estimate the total number of cysts within the hepatopancreas using a conservative lower confidence interval from our calculations of the average number of cysts per gram of tissue. We then multiplied this lower-bound cyst estimate by a mass constant (7.23869*10^–6^ g) for a single metacercaria, which was determined by obtaining an average cyst diameter (0.303 ± 0.089) from several (n = 15) *M*. *similis* metacercarial cysts, converting that diameter to a volume, and then multiplying that volume by a typical density (1.1 g/cm^3^) for most tissues (as used in [22] for multiple metazoan parasites including metacercariae; R. Hechinger, pers. comm.). This formula therefore provided an estimate of the total wet weight of metacercariae within the hepatopancreas. However, because our total hepatopancreas weight for the crab was a dry mass, we determined the ratio of the total cyst wet mass to the total hepatopancreas wet mass from all squashes and then multiplied this by the hepatopancreas dry mass, thus providing a conservative estimate of the total dry weight of metacercarial cysts within a crab’s hepatopancreas. For the gonad, we had a single squash (which captured much, if not all, the gonad tissue within a crab), so counts from this one squash were used to estimate metacercarial weight in the gonad as described for the hepatopancreas.

Additionally, some crabs were missing walking legs or chelipeds when they were assessed for physiology and this can skew HSI and GSI estimates by reducing overall body mass used to calculate these indices. We therefore accounted for missing limbs by obtaining dry weights (g) of a subset (n = 31) of crab limbs (walking legs and chelipeds) and then regressing limb weights with CW (walking legs: R^2^ = 0.582; cheliped: R^2^ = 0.751) to obtain a regression formula that we used to calculate and replace the weight of lost limbs. After all these adjustments and conversions were made, HSI and GSI were then regressed with cyst abundance to determine if infection intensity influenced these physiological indices.

### Calorimetry and Lipid Analyses

We determined the energy content of the hepatopancreas for the majority of the crabs (control = 18; 24-h = 10; 72-h = 12; 120-h = 10) using a 0.1g subsample of the hepatopancreas that was pressed into a firm pellet and combusted using a Parr 6725 semi-micro oxygen bomb calorimeter. This provided the mass-specific energy content; thus it was converted to the total energy of the hepatopancreas by multiplying it with the corrected hepatopancreas mass for each crab (where metacercariae mass was removed using our lower bound confidence interval of estimated metacercariae in the hepatopancreas; see methods above). In this analysis, we made the assumption that energy content of cyst tissue in the subsample was the same as crab tissue. We used a regression to determine any effects of cyst intensity on energy content in the hepatopancreas.

We also analyzed the bulk lipid content of the five most infected crabs (28,000 ± 17,000 cysts per g of hepatopancreas tissue) and the five least infected crabs (zero infection) to look for any differences related to infection status. We performed bulk lipid extraction on a 1g subsample of the hepatopancreas of each of these crabs, using a modified [64–65] method in which chloroform is replaced with hexanes [65]. The lipid contribution of these two groups (infected versus uninfected) were compared using a t-test. Because there was no difference in lipid content of these two extreme groups of crabs (see Results), lipids in the remaining crabs were not analyzed.

### Immune Activation Assay

To assess whether exposure to trematode cercariae stimulated a cellular immune response in crab hosts, a separate group of control and exposed crabs were bled approximately 72h after trematode cercariae exposure and then again ~4 weeks after exposure. In each crab, undiluted hemolymph was withdrawn from under the unsclerotinized membrane of a single walking leg from 9 control and 10 exposed crabs using a sterile 23 gauge needle and a cold 10mL disposable syringe and deposited into a microcentrifuge tube on ice. Twenty microliters of hemolymph were immediately pipette-mixed into 100uL of cold marine anticoagulant solution (per [66]) and then distributed to two wells of a KOVA Glasstic gridded slide (Hycor), where they were photographed at 40x on a compound microscope for cell counts. We counted all hemocytes in a 0.33mm square (or 0.66mm square for lower-density samples) grid for six replicate pictures per crab to determine the mean density of hemocytes per μL of diluted hemolymph. We compared the density of circulating hemocytes in control and experimental groups separately for the 72-h and 4-week post-exposure groups using t-tests. Differences in the time hemolymph was collected (and of tidal cycle, which can influence hemocyte densities; [67]) for the two sampling periods (72h and 4 weeks) prevented us from directly comparing circulating hemocyte densities between the time periods.

## Results

Below, we present our results for each experiment. We tested crab sex as a potential explanatory factor for all experiments, but in our exploratory analyses, we found few differences between the sexes, thus results below represent pooled data for males and females (unless otherwise stated).

### Infection Induction Experiment

A subset of *Littorina* spp. exposure snails (n = 200) were dissected. Twenty-nine percent of these were infected with trematodes, and 9% were specifically infected with *Microphallus similis*. Each infected snail can contain hundreds of cercariae, and our cercarial emergence data (S1 Fig) demonstrated a continual supply of *M*. *similis* in the aquaria, especially in the mornings after dessicated snails had been added.

**Fig 2:**
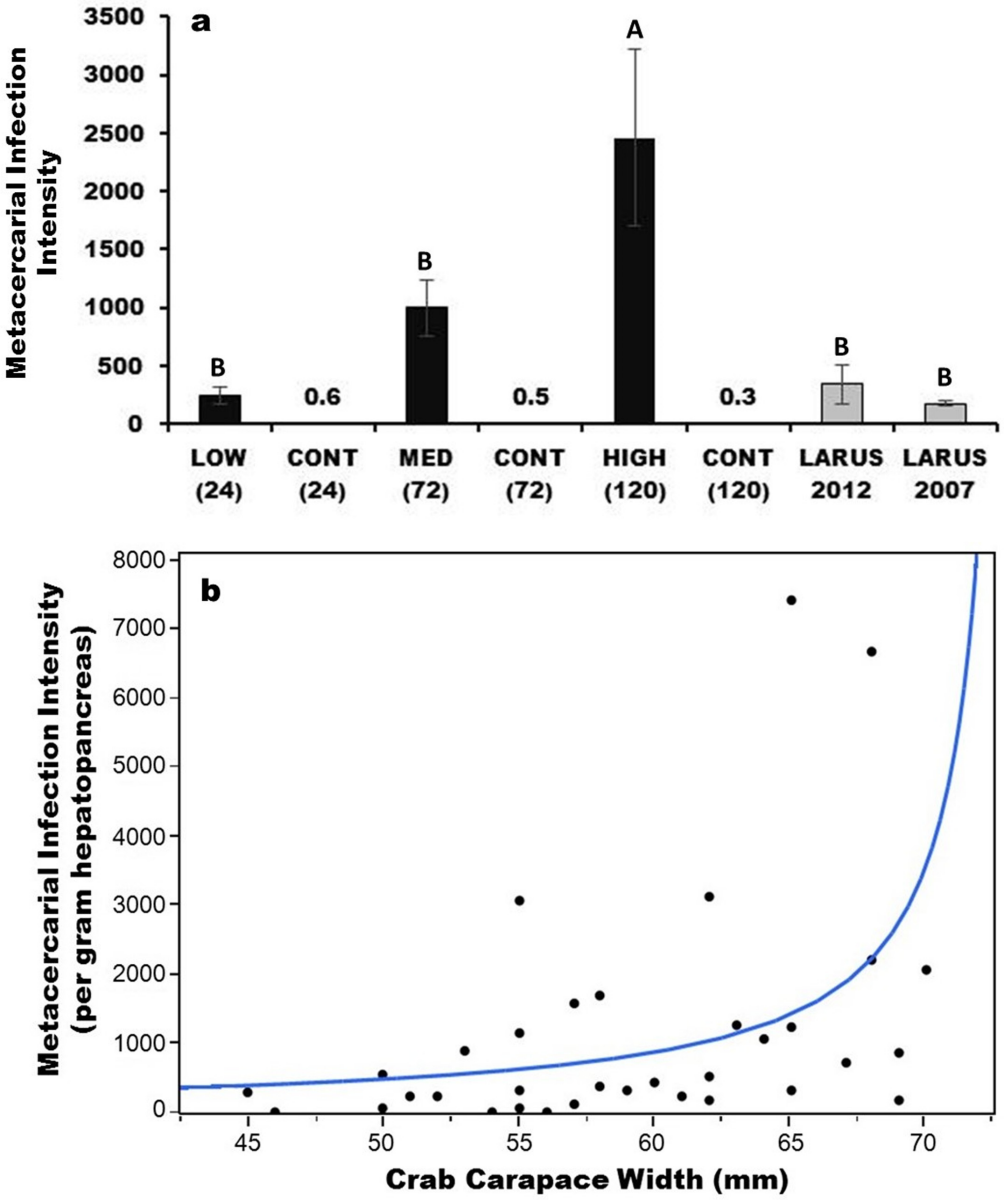
Average (± SE) infection intensity of metacercarial cysts in hepatopancreas, gonad, and thoracic ganglion tissues in each treatment and naturally at Appledore Island, ME and metacercarial cyst intensity per gram hepatopancreas tissue by crab carapace width (mm). (a) Differences in capitalized letters above the bars indicate a significant difference in infection intensity (p<0.05) of counted cysts. Numbers above the control bars indicate the average infection intensities per control (all less than 1). LOW (24) and CONT (24) refer to the 24 hour infection treatment and its associated control; MED (72) and CONT (72) refer to the 72 hour infection treatment and its associated control; HIGH (120) and CONT (120) refer to the 120 hour infection treatment and its associated control; ‘LARUS’ refers to naturally infected crabs collected at Larus Ledge in 2007 and 2012 on Appledore Island, Isles of Shoals, ME. (b) There is a significant exponential relationship between crab size (CW) and cyst intensity per gram hepatopancreas.

We found significant differences among infection intensities and exposure times in all tissues (hepatopancreas, gonad, and thoracic ganglion) across the 70 crabs (p<0.001), and a post-hoc Tukey’s test revealed a significantly (p<0.01) greater infection intensity for the 120-h exposure treatment compared to the 24-h and 72-h treatments, as well as all three controls; in addition, natural infections from Appledore Island in 2007 and 2012 were not significantly different from either the 24-h or 72-h treatments but were significantly lower than the 120-h treatment (Fig 2A). We also found crab carapace width (CW) to significantly correlate with infection intensity per gram of hepatopancreas tissue when an exponential distribution was modeled (AICc = 555; p = 0.0006, versus a linear distribution AICc = 619; p = 0.0163); i.e., larger crabs contained exponentially more cysts per gram of hepatopancreas than did smaller crabs (Fig 2B).

In many of the 72-h and 120-h crabs, we found numerous cysts in the thoracic ganglion, the crab’s nerve center. However, cyst abundance was positively correlated in pairwise comparisons of tissue types for all crabs (ganglion and hepatopancreas correlation = 0.776; p<0.0001; ganglion and gonad correlation = 0.387; p = 0.0007; hepatopancreas and gonad correlation = 0.368; p = 0.0013); as such, we pooled metacercarial counts into a single value for cyst infection intensity for the behavioral and physiological analyses below (unless otherwise stated). Finally, our analysis of the space cysts exploit in a heavily infected crab (>7000 counted cysts) suggested they could occupy 18–54% (36% ± 18%) of a crab’s hepatopancreas.

### Righting Response Trials

**Fig 3:**
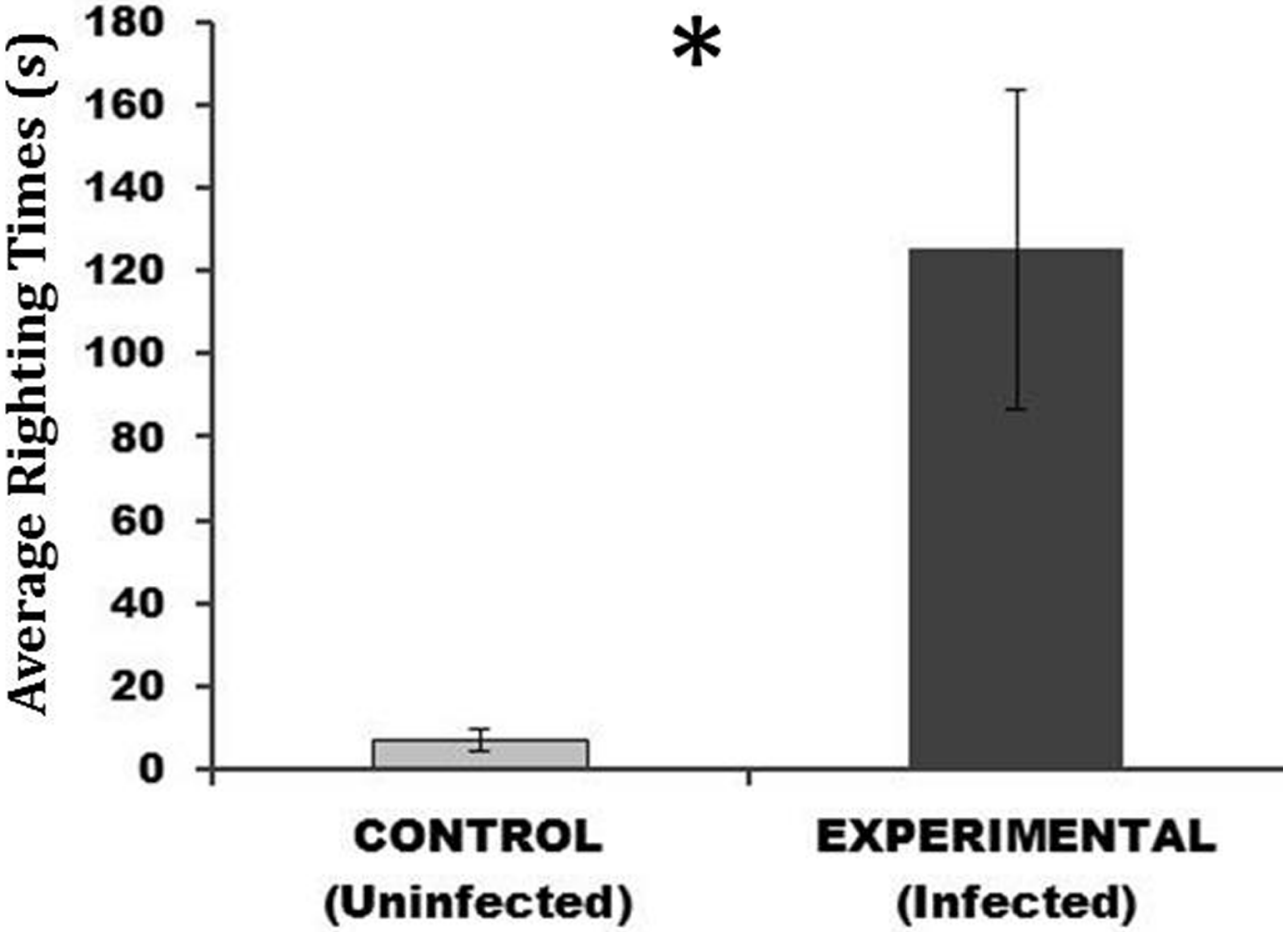
Average (± SE) righting response time (seconds) for uninfected and infected crabs from the 72h (medium) treatment. This demonstrates the average time it took for crabs to right themselves after being placed on their dorsal side. These trials took place two hours after the 72h induction experiment ended, shortly after cercarial penetration of crab tissues.

We found a significant difference (p = 0.002) in the average righting response time of infected versus uninfected crabs (Fig 3). The average (±SE) righting time in seconds for infected individuals (125 ± 38) was 17 times higher than the average righting time for uninfected individuals (7 ± 3).

### Behavioral Trials after Four Week Incubation

**Fig 4:**
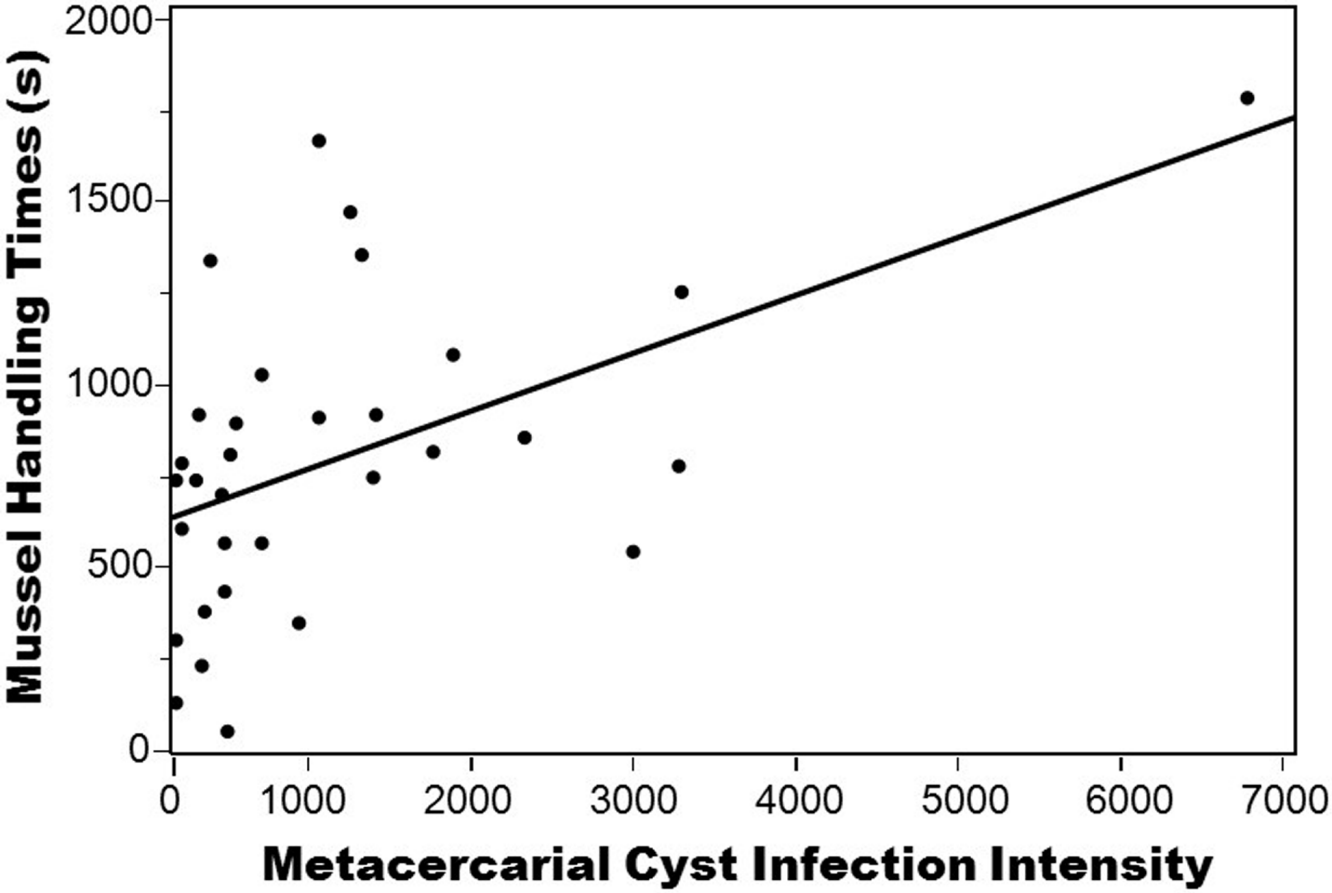
Regression of mussel handling time by metacercarial cyst intensity. Metacercarial infection intensity represents actual counts. The regression remains significant with (R^2^ = 0.238; p<0.001) or without (R^2^ = 0.123; p = 0.005) the crab with 6500 cysts.

Regressions of cyst intensity with the various behaviors recorded in the ethogram (providing a relative understanding of the types and magnitudes of behaviors a crab performed in the thirty minute period) found no significant or strong correlations for individual behaviors or when behaviors were pooled into behavior types (shelter-use, foraging, active, inactive) (see S1 Table for a list of behaviors and regression statistics). Moreover, independently timed behaviors also demonstrated no correlation with cyst intensity, except for a significant positive correlation between average mussel handling time and infection intensity (R^2^ = 0.238; p<0.001) (Fig 4), and this correlation remained intact even when a possible outlier (a crab with ~6500 cysts) was removed (R^2^ = 0.123; p = 0.005). A principle components multivariate analysis using a correlation matrix (S4 Fig) also demonstrated mussel handling time to be the strongest correlative variable (0.488) of crab behavior with cyst intensity.

### HSI and GSI, Calorimetry, and Lipid Analyses

**Fig 5:**
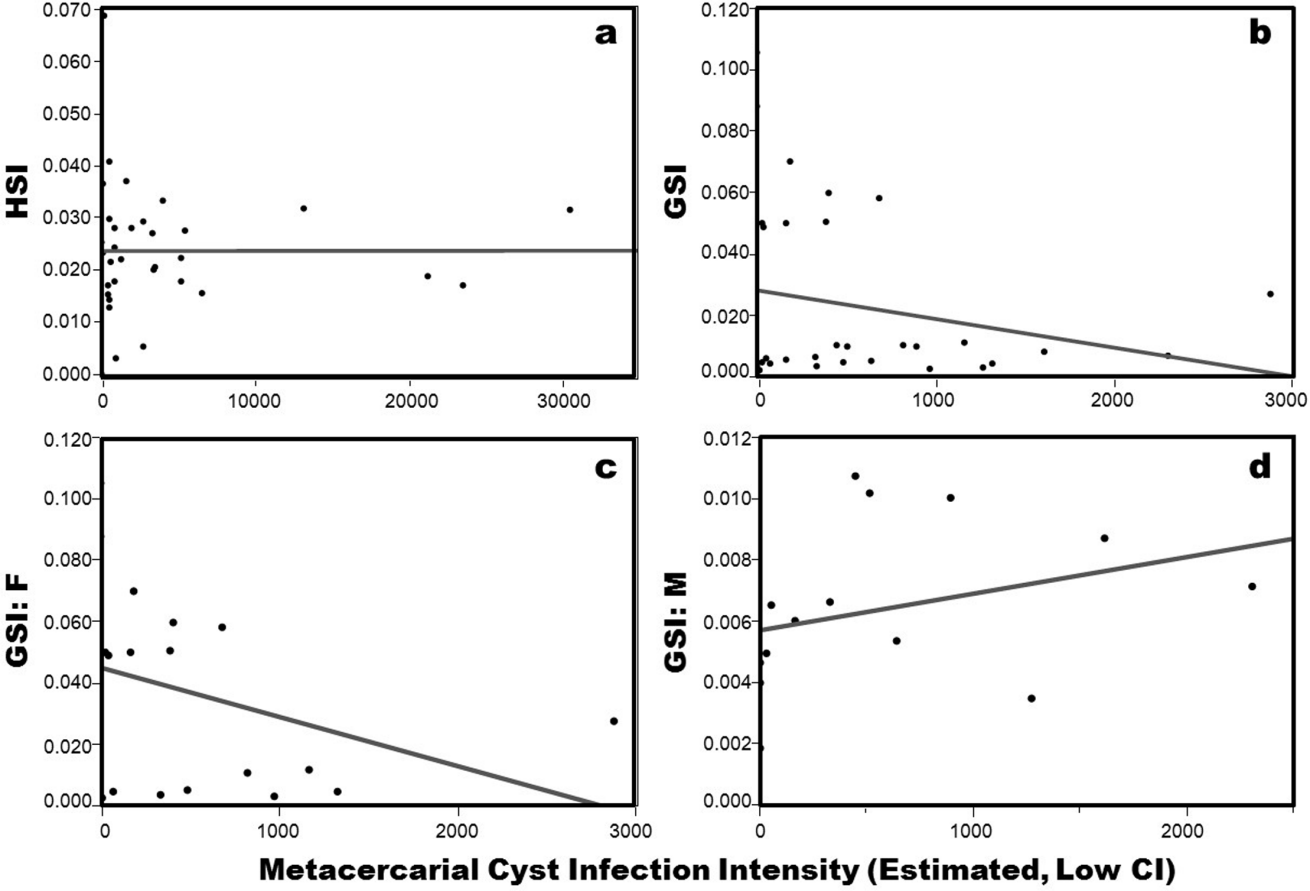
Hepatosomatic index (HSI) and gonadosomatic index (GSI) analyses by cyst intensity (estimated metacercarial abundance). Panel ‘a’ represents HSI for all crabs combined; ‘b’ represents GSI for all crabs combined; ‘c’ represents GSI for females only; ‘d’ represents GSI for males only. Cyst intensity for HSI is lower bound estimated hepatopancreas metacercarial cyst abundance, and cyst intensity for GSI is lower bound estimated gonad metacercarial cyst abundance.

There was no correlation between HSI and cyst intensity (R^2^ = 0.000; p = 0.996) (Fig 5A). For GSI, there was no correlation with cyst intensity when females and males were pooled (R^2^ = 0.052, p = 0.210) or separated (F: R^2^ = 0.125, p = 0.150; M: R^2^ = 0.099, p = 0.273) (Fig 5B–5D).There was also no significant effect of cyst intensity (R^2^ = 0.003, p = 0.727) on resulting calorimetry of the hepatopancreas. Moreover, in our lipid analysis of the five most infected versus five least infected crabs, there was no significant differences in the average % lipid content between the two groups (p = 0.280).

### Immune Activation

**Fig 6:**
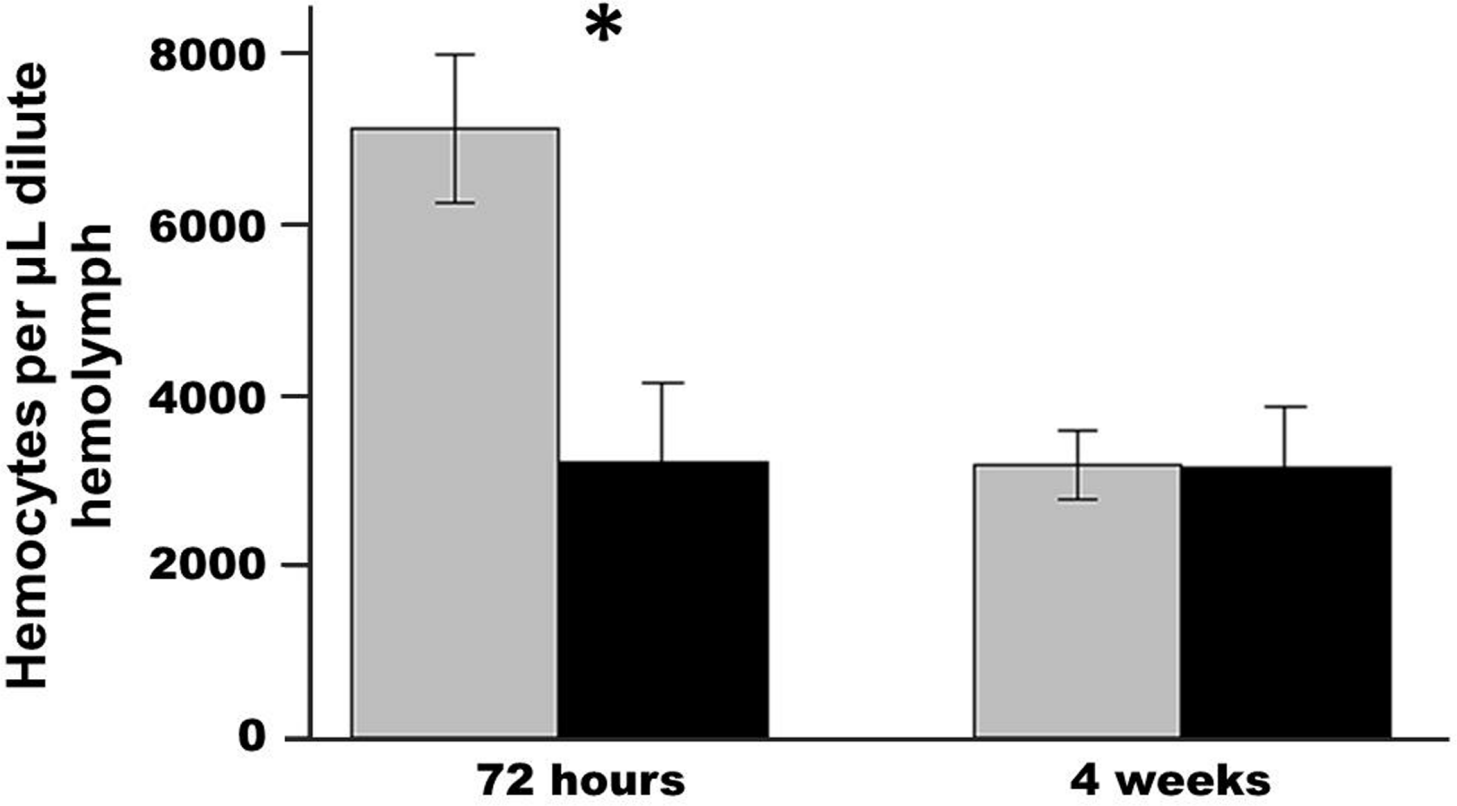
Average (± SE) hemocyte counts in crabs 72 hours after exposure and after the four week incubation period. The grey bar is the control and the black bar is the experimental treatment showing circulating hemocytes per μL hemolymph from blood drawn for exposed and control crabs for two exposure periods. *refers to a significant difference (p<0.05) between the treatments.

Exposure to trematode cercariae significantly depressed the density of circulating hemocytes in crabs sampled immediately after the 72-h exposure relative to controls (p<0.008; Fig 6). However, this trend did not persist for crabs sampled 4 weeks after exposure (p = 0.97).

## Discussion

While prior investigations of host-trematode systems have found numerous intermediate hosts to be susceptible to trematode-mediated behavioral manipulations (e.g., [25]) and physiological impairment [33], we found little evidence for lasting physiological and behavioral impacts in parasitized crabs four weeks after experimental infection. In particular, none of the physiological indices (HSI or GSI), nor lipid or calorimetry analyses, demonstrated any significant correlations with cyst intensity. Specific to reproduction, a prior investigation of parasite influence in native and non-native populations of *C*. *maenas* also found no effect of microphallid trematode infection on a reproduction index in the crab [68]. Likewise, behavioral experiments demonstrated little effect of *M*. *similis* infection intensity on crab foraging, shelter-use, or conspicuousness four weeks after experimental infection, even though it was common to observe hundreds of cysts in the crab’s thoracic ganglia (nerve center) in highly infected individuals. Similarly, a different decapod species, the Dungeness crab (*Cancer magister*), demonstrated little behavioral response to metacercariae encysted within its nervous system (including the ganglia) [69]. However, our study did uncover a significant positive correlation between cyst intensity and crab handling time of mussels (i.e., the more infected a crab was, the longer it took (on average) to crush and consume mussels), and a multivariate PCA analysis confirmed this to be the strongest correlative variable with cyst intensity among all investigated behaviors. Interestingly, though handling time increased with infection intensity, we found no effect of intensity on the number of mussels consumed during our 30-minute observations (though a shorter observation period may have revealed an influence). In many mesopredators, longer prey handling times have been shown to enhance risk of predation, competition, wave dislodgement, among other biotic and abiotic factors in intertidal marine systems [70–73]. Moreover, trematode infection itself can influence host foraging; for example, due to enhanced handling time of larger prey items as a result of trematode parasitism, infected three-spined sticklebacks were observed to switch to smaller prey [74]. Moreover, infected individuals of the common periwinkle snail, *Littorina littorea*, demonstrated lower consumption rates of ephemeral algae compared to uninfected individuals, and this conferred a positive indirect effect on the prey, which had significantly greater percent cover in experimental plots compared to controls with uninfected snails [38]. Interestingly, an increase in mussel handling time has also been observed in *C*. *maenas* in its native range as a result of exposure to an environmental contaminant [75], and this could suggest analogous responses to different types of stressors.

Though our physiological and behavioral experiments demonstrated little effect of *M*. *similis* on *C*. *maenas* after the four week ‘incubation’ period, we found a strong signal of immune system activation in experimental crabs shortly after trematode exposure. In particular, we observed significantly fewer hemocytes (per μL) in hemolymph from exposed crabs compared to control crabs just 72 hours after exposure to cercariae. This drop in hemocyte density after exposure likely resulted from the recruitment of hemocytes to encysting metacercaria, as seen in histological studies of other trematode species in *C*. *maenas* and in other crab intermediate hosts [33, 76–77]. While this induced effect did not appear to exert a long term energy cost (i.e., there was no effect of infection on our measures of energy storage after four weeks), it is possible that it resulted in a shorter term energy expenditure as hemocytes were consumed and replenished during early stages of cercarial penetration [78–79]. Remarkably, this signal of immune activation in infected crabs had disappeared by four weeks, and the two groups showed similar circulating hemocyte densities. Likewise, a trematode infection experiment of an amphipod intermediate host found parasite-induced mortality to be highest during the first two days after exposure (i.e., while cercariae were actively penetrating and migrating through the host) and declined later on; mortality was also found to be intensity dependent [80]. It is possible that hemocyte production or changes in circulation are not particularly expensive in terms of resource diversion, as large fluctuations in circulating hemocyte densities have been associated with environmental contaminants, tidal fluctuations, and molt cycle in other decapods as well as in *C*. *maenas* [67, 81]. Overall, our finding of a significant but ephemeral negative impact of trematode exposure seems to suggest that the primary pathology for the second intermediate host occurs early in the infection process and declines after the trematode successfully encysts.

A second effect of *M*. *similis* infection shortly after cercarial penetration was a significantly slower righting response time in infected crabs, whereby infected individuals demonstrated greater lethargy than uninfected individuals. We also anecdotally noted enhanced lethargy in infected versus control crabs in the 120-h (high) infection treatment just after the induction experiment; however, these lethargic behaviors seemed to disappear over time, and by four weeks, heavily infected crabs qualitatively behaved similarly to control crabs. Slower righting response times due to environmental conditions like temperature, hypoxia, and chemical toxins have been observed in several species of crabs (e.g., [82–84]), and in Norway lobsters, infection by a parasitic dinoflagellate resulted in lower swimming capacity in infected versus uninfected individuals [85]. Overall, slower response times and increased lethargy could be detrimental to afflicted individuals that are less able to flee from predators, thereby increasing their predation risk [74, 86].

Another noteworthy aspect of our study was that we were able to clearly and significantly establish a gradient of infection based on exposure time to infected snails, where actual cyst counts ranged from approximately 20 to 7000 cysts (total estimates were 50 to >40,000 cysts) in the 24-h and 120-h treatments, respectively. In fact, we calculated that a heavily infected crab could have as much as 53% of its internal hepatopancreas tissue taken up by *M*. *similis* cysts. Even with such a significant space appropriation, the marginal lasting effects of cyst intensity we found in infected crabs may be partly attributed to a slowing of cyst growth at four weeks associated with a reduction in energy extraction that would have been much stronger during early stages of active cyst growth; i.e., [59] found *M*. *similis* cyst size to increase from 0.05 to 0.55 mm during cyst maturation, while in our study, we calculated cyst diameters at four weeks to be on average about 0.30 (±0.09 SD) mm. Thus, at this point of maturation, cysts will continue to grow but likely not as rapidly as early stages, which may also help explain why we observed a stronger influence of *M*. *similis* shortly after infection compared to four weeks later.

In our study, we also revealed a significant positive correlation between crab size and cyst intensity, and this correlation was stronger when fit to an exponential rather than a linear model. Positive correlations between size and infection intensity are common in trematode intermediate hosts, and a plausible explanation for this pattern is time; i.e., larger/older individuals have had more time to accrue infections [32, 57, 87–88]. In our study, however, time is not a relevant factor since the experiment was carried out over a <5 day period. While larger hosts naturally have greater space for more cysts, the exponential relationship we uncovered seems to suggest other factors may be involved. For example, host chemical cues are an important part of the infection process and may influence cercarial host-finding behavior [89–90] resulting in greater infection in certain size classes of intermediate hosts [23]. For *C*. *maenas*, larger crabs likely elicit the strongest chemical cues, possibly attracting more cercariae and enhancing infection intensity in larger individuals. Alternatively (or in addition to), cercariae could respond to cues from one another and demonstrate aggregation behaviors. Aggregation behaviors have been observed in many trematode species, and chemical communication among trematode larval stages is a potential mechanism for aggregation [74, 91–92]. In fact, aggregation may be adaptive due to the chance nature of infection; i.e., many cercariae die before they can infect a host [93], thus those that do successfully infect a second-intermediate host may aggregate to alleviate possible Allee effects when reaching the definitive host [92]. However, high intensities of infection with trematode metacercariae have also been correlated with smaller metacercarial size and lower fecundity in resulting adult worms, suggesting a density-dependent fitness cost to the parasite [30].

In conclusion, our study represents the first endeavor exploring both physiological and behavioral impacts of trematode parasitism in *C*. *maenas*, a highly successful global invader. Trematodes are a parasite group frequently associated with substantial host impacts, including tissue damage, reproductive damage, and parasite-increased trophic transmission [25, 33–35, 42]. However, our study revealed few significant effects of *M*. *similis* cyst intensity on *C*. *maenas* physiology or behavior, except at very early stages of infection. Once crabs surpassed those early stages, they seemed to “recover” and show few differences from uninfected crabs. Thus, even though *C*. *maenas* has failed to escape one of its most prevalent parasites in eastern North America [44], even high intensity infections of *M*. *similis* appear to have minimal long-term physiological or behavioral influences on the crab. This may suggest that, in some cases, the transfer or acquisition of certain parasites in marine NIS may do little to hinder an invasive host population, like green crabs in eastern North America. Further investigations throughout multiple non-native ranges where NIS are infected by few to no parasites can provide a greater understanding of the influence that parasite escape and parasite release may be having on worldwide populations of NIS, in general, and *C*. *maenas* specifically [18, 68].

## Supporting Information

S1 Fig Cercarial emergence counts for *Microphallus similis* only (A) and all cercariae (B). These counts were performed every 12h during the induction experiment for the two batches of snails (see methods) for the low treatment (diamond = 24 hour exposure), the medium treatment (square = 72 hour exposure), and the high treatment (triangle = 120 hour exposure). Each point represents five 1 ml replicate samples. ‘M’ stands for morning. Control treatments are not shown because all control aquaria had 0 cercariae for every sampling point.
(TIF)

S2 Fig Experimental arenas for behavioral trials. A schematic of the experimental arena set-up including the shelter (*Fucus*-covered rock) and the food item (five mussels) is shown in (A). The actual set-up is shown in (B), including the two acclimation arenas on the left and the two experimental arenas on the right with video cameras mounted above the experimental arenas such that the whole arena could be recorded. After each trial, the video camera mount was moved to record crabs that had been acclimating in the other two arenas. Red lighting was used to illuminate the arenas for recording.
(TIF)

S3 Fig Naturally acquired (old) versus experimentally-induced (new) *Microphallus similis* metacercarial cysts in infected *Carcinus maenas*. The image of the old cyst comes from a naturally infected crab at Appledore Island; old cysts are distinguished by a thick cyst wall. The new cyst image comes from an experimentally infected crab in a four-week old infection; newer cysts have a very thin cyst wall. Images taken by AMHB on a compound microscope at 4x magnification.
(TIF)

S4 Fig Principle Components Analysis (PCA) of Behavior Data. Analysis is based upon a correlation matrix from the behavioral ethogram data. 45% of the variation is accounted for in PC1 and PC2. The highest correlation with cyst intensity (total cysts) was with mussel handling time.
(TIF)

S1 Table Statistical results of video analyses for behavioral trials after four-week incubation. **Individual regressions with cyst intensity (total cyst abundance per crab) are reported for each of the recorded behaviors in the ethogram across all crabs**. The ethogram data provide a relative understanding of the types and magnitudes of behaviors that crabs performed every 30s over the thirty minute recording period. Behaviors were independently assessed and also grouped into different categories, including all shelter related behaviors (walking/climbing on shelter, on shelter, under shelter, next to shelter), all foraging related behaviors (handling, cracking, and opening mussels and consuming mussels), active/conspicuous behaviors (walking, climbing walls, walking/climbing shelter, handling and consuming mussels), and inactive behaviors (standing, on shelter, under shelter, next to shelter). Timed behaviors (in seconds) are also recorded. These were behaviors that were independently timed during video analysis to assess the potential effect of cyst intensity on the total amount of time crabs spent at the shelter, the total amount of time it took for crabs to encounter a mussel (i.e., search time), and the handling time of mussels averaged across all handled mussels (out of 5 provided)—this latter analysis is listed twice to demonstrate the effect of removing a possible outlier (a crab with 6500 cysts) (see Fig 4). Finally, the proportion of mussels consumed (out of 5 provided) was also regressed with cyst intensity. Significant regressions are denoted by a (*) and are bolded.
(PDF)

S1 File Ethogram of crab behaviors during each 30 minute trial. Column 1 includes each 30 second time points. Behaviors were then divided into different types depending on shelter or foraging. Any behaviors observed at each 30 second time point were marked and then the number of times these behaviors occurred within 30 minutes were tallied at the bottom.
(PDF)
