# Supplementary figures and images for: Assessing the Effects of Trematode Infection on Invasive Green Crabs in Eastern North America
Source: PLoS One. 2015 Jun 1;10(6):e0128674. doi: 10.1371/journal.pone.0128674 (PMC4451766; doi:10.1371/journal.pone.0128674)

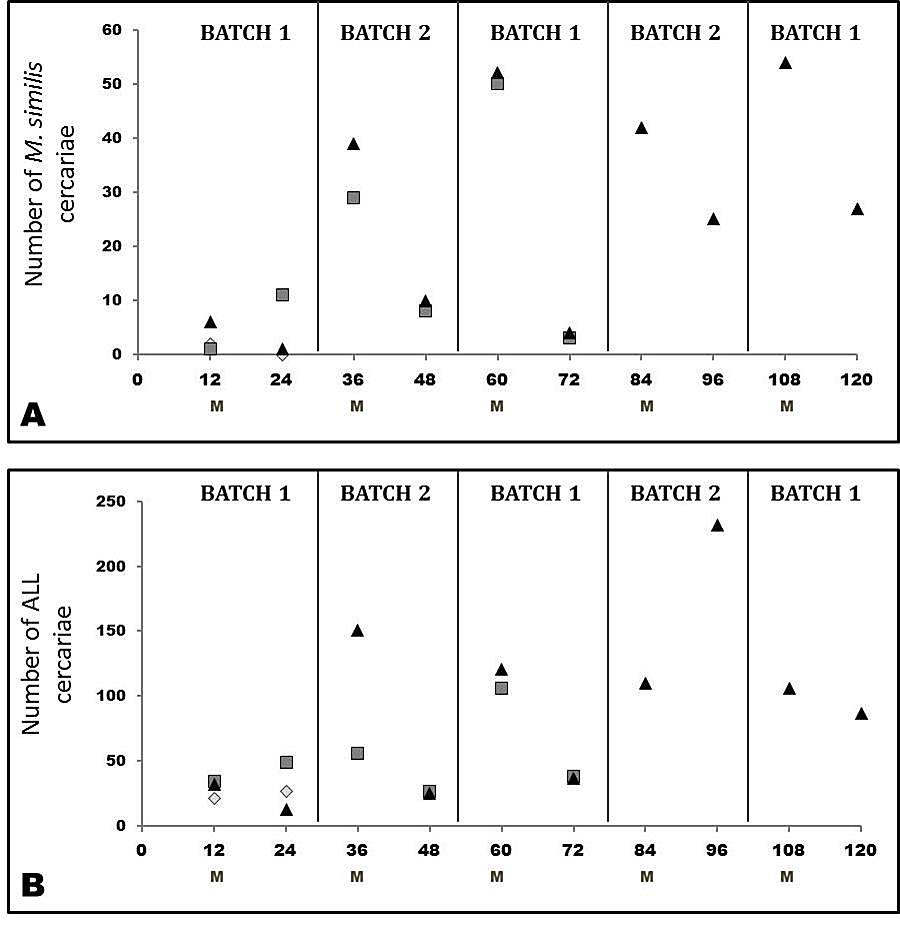

Supplement: S1 Fig — These counts were performed every 12h during the induction experiment for the two batches of snails (see methods) for the low treatment (diamond = 24 hour exposure), the medium treatment (square = 72 hour exposure), and the high treatment (triangle = 120 hour exposure). Each point represents five 1 ml replicate samples. ‘M’ stands for morning. Control treatments are not shown because all control aquaria had 0 cercariae for every sampling point. (TIF) [file pone.0128674.s001.tif]

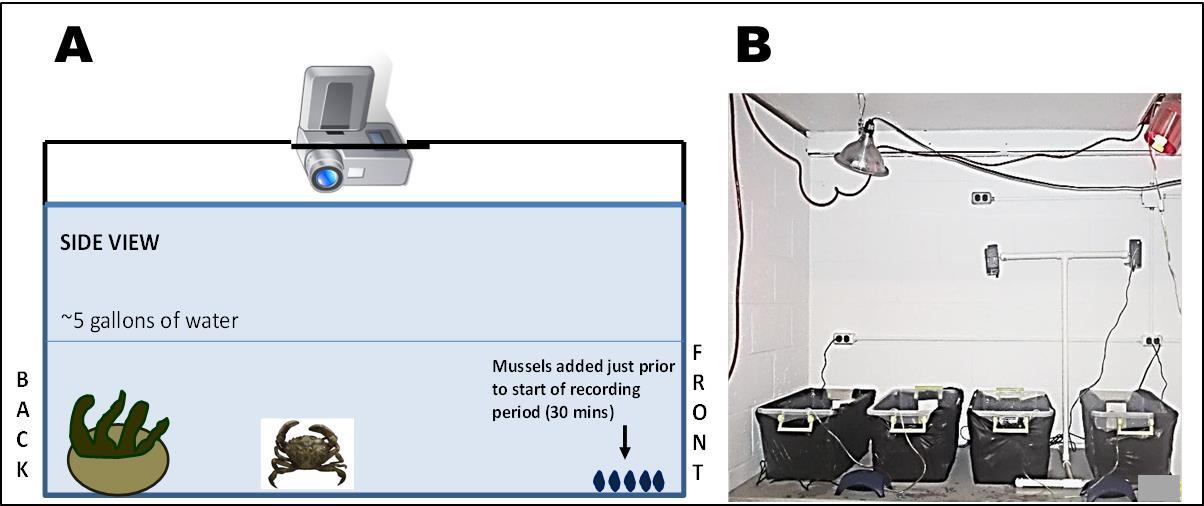

Supplement: S2 Fig — A schematic of the experimental arena set-up including the shelter (Fucus-covered rock) and the food item (five mussels) is shown in (A). The actual set-up is shown in (B), including the two acclimation arenas on the left and the two experimental arenas on the right with video cameras mounted above the experimental arenas such that the whole arena could be recorded. After each trial, the video camera mount was moved to record crabs that had been acclimating in the other two arenas. Red lighting was used to illuminate the arenas for recording. (TIF) [file pone.0128674.s002.tif]

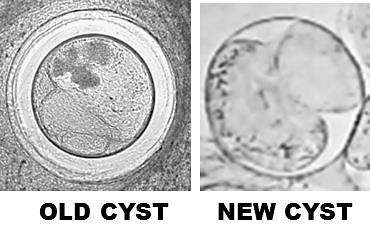

Supplement: S3 Fig — The image of the old cyst comes from a naturally infected crab at Appledore Island; old cysts are distinguished by a thick cyst wall. The new cyst image comes from an experimentally infected crab in a four-week old infection; newer cysts have a very thin cyst wall. Images taken by AMHB on a compound microscope at 4x magnification. (TIF) [file pone.0128674.s003.tif]

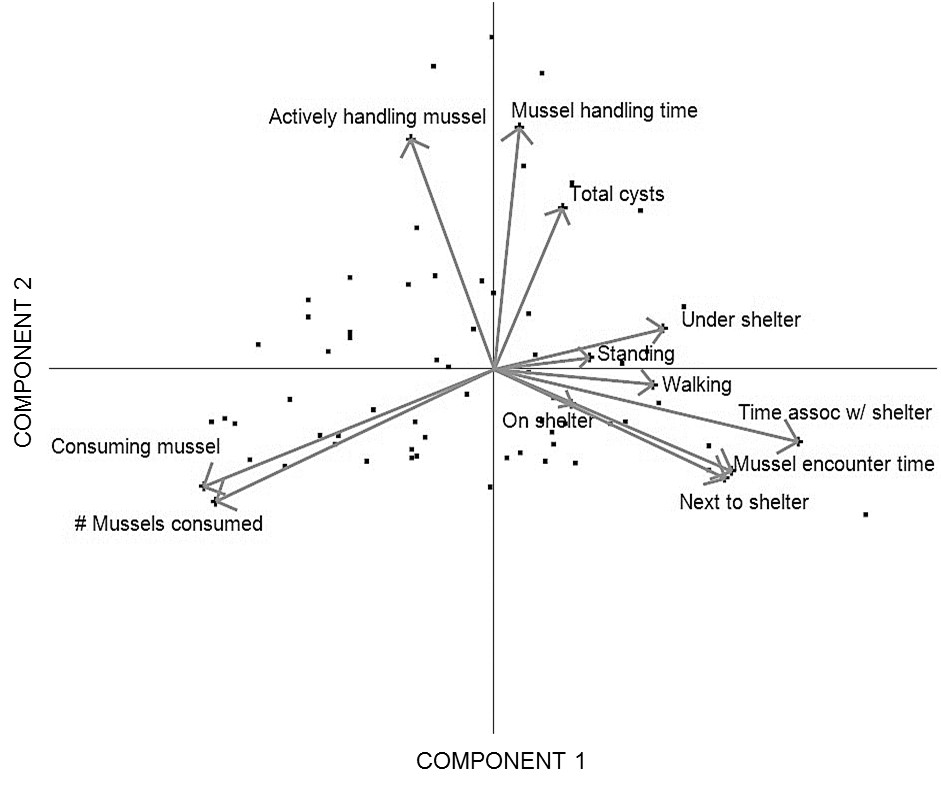

Supplement: S4 Fig — Analysis is based upon a correlation matrix from the behavioral ethogram data. 45% of the variation is accounted for in PC1 and PC2. The highest correlation with cyst intensity (total cysts) was with mussel handling time. (TIF) [file pone.0128674.s004.tif]
