# Supplementary material for: Assessing the Effects of Trematode Infection on Invasive Green Crabs in Eastern North America
Source: PLoS One. 2015 Jun 1;10(6):e0128674. doi: 10.1371/journal.pone.0128674 (PMC4451766; doi:10.1371/journal.pone.0128674)
Supplement: S1 Table — Individual regressions with cyst intensity (total cyst abundance per crab) are reported for each of the recorded behaviors in the ethogram across all crabs. The ethogram data provide a relative understanding of the types and magnitudes of behaviors that crabs performed every 30s over the thirty minute recording period. Behaviors were independently assessed and also grouped into different categories, including all shelter related behaviors (walking/climbing on shelter, on shelter, under shelter, next to shelter), all foraging related behaviors (handling, cracking, and opening mussels and consuming mussels), active/conspicuous behaviors (walking, climbing walls, walking/climbing shelter, handling and consuming mussels), and inactive behaviors (standing, on shelter, under shelter, next to shelter). Timed behaviors (in seconds) are also recorded. These were behaviors that were independently timed during video analysis to assess the potential effect of cyst intensity on the total amount of time crabs spent at the shelter, the total amount of time it took for crabs to encounter a mussel (i.e., search time), and the handling time of mussels averaged across all handled mussels (out of 5 provided)—this latter analysis is listed twice to demonstrate the effect of removing a possible outlier (a crab with 6500 cysts) (see Fig 4). Finally, the proportion of mussels consumed (out of 5 provided) was also regressed with cyst intensity. Significant regressions are denoted by a (*) and are bolded. (PDF) [file pone.0128674.s005.pdf]

Assessed Behaviors	Regression with cyst intensity	
	R^2	p
Behaviors recorded in Ethogram		
Walking	0.0131	0.3533
Standing	0.0159	0.3060
Climbing walls	0.0096	0.4282
Walking/climbing on shelter	0.0199	0.2512
On shelter	0.0052	0.5624
Under shelter	0.0444	0.0843
Next to shelter	0.0001	0.9125
All shelter related behaviors	0.0148	0.3232
Handling, cracking, opening mussels	0.0078	0.4755
Consuming mussels	0.0208	0.2404
All foraging related behaviors	0.0014	0.7618
Active/conspicuous behaviors	0.0151	0.3177
Inactive behaviors	0.0225	0.2217
Timed behaviors (seconds)		
Total time spent in association with shelter	0.0160	0.3044
Mussel encounter time (search time)	0.0001	0.9304
Mussel handling time (avg)	**0.2378**	**<0.0001***
Mussel handling time (avg) (possible outlier removed)	**0.1230**	**0.0050***
Mussels consumed after 30 min		
Proportion of mussels consumed (out of 5)	0.0150	0.3123
