# Supplementary material for: Assessing the Effects of Trematode Infection on Invasive Green Crabs in Eastern North America
Source: PLoS One. 2015 Jun 1;10(6):e0128674. doi: 10.1371/journal.pone.0128674 (PMC4451766; doi:10.1371/journal.pone.0128674)
Supplement: S1 File — Column 1 includes each 30 second time points. Behaviors were then divided into different types depending on shelter or foraging. Any behaviors observed at each 30 second time point were marked and then the number of times these behaviors occurred within 30 minutes were tallied at the bottom. (PDF) [file pone.0128674.s006.pdf]

TIME (min:sec)	Crab ID: ________________________									
	OTHER			ASSOCIATED WITH SHELTER				ASSOCIATED WITH MUSSELS		
	Walking	Standing still	Climbing up walls / corners of aquarium	Actively walking or climbing on shelter	On top of shelter	Under the shelter	Next to the shelter	Actively handling mussel (s)	Cracking / breaking into mussel (s)	Consuming mussels
0:30										
1:00										
1:30										
2:00										
2:30										
3:00										
3:30										
4:00										
4:30										
5:00										
5:30										
6:00										
6:30										
7:00										
7:30										
8:00										
8:30										
9:00										
9:30										
10:00										
10:30										
11:00										
11:30										
12:00										
12:30										
13:00										
13:30										
14:00										
14:30										
15:00										
15:30										
16:00										
16:30										
17:00										
17:30										
18:00										
18:30										
19:00										
19:30										
20:00										
20:30										
21:00										
21:30										
22:00										
22:30										
23:00										
23:30										
24:00										
24:30										
25:00										
25:30										
26:00										
26:30										
27:00										
27:30										
28:00										
28:30										
29:00										
29:30										
30:00										
TOTALS										
